# Monocyte-derived dendritic cells loaded with a mixture of apoptotic/necrotic melanoma cells efficiently cross-present gp100 and MART-1 antigens to specific CD8^+ ^T lymphocytes

**DOI:** 10.1186/1479-5876-5-19

**Published:** 2007-04-20

**Authors:** Erika M von Euw, María M Barrio, David Furman, Michele Bianchini, Estrella M Levy, Cassian Yee, Yongqing Li, Rosa Wainstok, José Mordoh

**Affiliations:** 1 Fundación Instituto Leloir, Patricias Argentinas 435 (1405), Buenos Aires, Argentina; 2 Centro de Investigaciones Oncológicas – FUCA, Cramer 1180 (1426), Buenos Aires, Argentina; 3 Fred Hutchinson Cancer Research Centre, Clinical Research Division, 1100 Fairview Avenue N., D3-100, Seattle, Washington 98109-1024, US; 4 Departamento Química Biológica. Facultad de Ciencias Exactas y Naturales, Universidad de Buenos Aires, Buenos Aires, Argentina

## Abstract

**Background:**

In the present study, we demonstrate, in rigorous fashion, that human monocyte-derived immature dendritic cells (DCs) can efficiently cross-present tumor-associated antigens when co-cultured with a mixture of human melanoma cells rendered apoptotic/necrotic by γ irradiation (Apo-Nec cells).

**Methods:**

We evaluated the phagocytosis of Apo-Nec cells by FACS after PKH26 and PKH67 staining of DCs and Apo-Nec cells at different times of coculture. The kinetics of the process was also followed by electron microscopy. DCs maturation was also studied monitoring the expression of specific markers, migration towards specific chemokines and the ability to cross-present *in vitro *the native melanoma-associated Ags MelanA/MART-1 and gp100.

**Results:**

Apo-Nec cells were efficiently phagocytosed by immature DCs (iDC) (55 ± 10.5%) at 12 hs of coculture. By 12–24 hs we observed digested Apo-Nec cells inside DCs and large empty vacuoles as part of the cellular processing. Loading with Apo-Nec cells induced DCs maturation to levels achieved using LPS treatment, as measured by: i) the decrease in FITC – Dextran uptake (iDC: 81 ± 5%; DC/Apo-Nec 33 ± 12%); ii) the cell surface up-regulation of CD80, CD86, CD83, CCR7, CD40, HLA-I and HLA-II and iii) an increased *in vitro *migration towards MIP-3β. DC/Apo-Nec isolated from HLA-A*0201 donors were able to induce >600 pg/ml IFN-γ secretion of CTL clones specific for MelanA/MART-1 and gp100 Ags after 6 hs and up to 48 hs of coculture, demonstrating efficient cross-presentation of the native Ags. Intracellular IL-12 was detected in DC/Apo-Nec 24 hs post-coculture while IL-10 did not change.

**Conclusion:**

We conclude that the use of a mixture of four apoptotic/necrotic melanoma cell lines is a suitable source of native melanoma Ags that provides maturation signals for DCs, increases migration to MIP-3β and allows Ag cross-presentation. This strategy could be exploited for vaccination of melanoma patients.

## Background

Dendritic cells (DCs) are able to efficiently capture antigens (Ags) at their immature stage (iDC), process them and initiate immune responses upon interaction with lymphocytes [1]. The specialized functions of mature DCs (mDCs) are essential to start T cell mediated immunity since they can prime naïve T cells. As DCs take up and process Ags and in response to various stimuli [2-6] they undergo a process of maturation and express large amounts of human leukocyte Ags (HLA) – peptide complexes at their surface. To achieve their function, DCs must arrive into the lymph nodes in response to several chemoattracting signals that bind specific cell surface receptors expressed by DCs during the maturation process, such as CCR7 (CC chemokine receptor) [7]. Once DCs have arrived into secondary lymphoid organs, they can stimulate naïve T cells [8].

A common strategy used for vaccine preparation is to load DCs with exogenous peptides from tumor associated Ags (TAAs) on empty HLA class I molecules [9-11]. This approach, however, has the limitations of peptide restriction to a given haplotype and the induction of responses to only one or few defined Ags. In order to use a broader spectrum of known and yet unknown Ags for DCs loading, the approach of whole tumor cells is preferred. We and others have demonstrated that when murine DCs that had phagocytosed apoptotic B16 melanoma cells were used as vaccines, they were able to induce an effective, long-term protection against challenge with live B16 cells [12,13].

Since the induction of CD8^+ ^cytotoxic T lymphocytes (CTLs) appears to play a central role in the process of protective immunity, only cross-presentation of tumor Ags acquired from whole tumor cells would confer effective antitumor immunity. Several authors have demonstrated in murine models [14,15] and in humans [16]. that when DCs engulf apoptotic cells, Ags can be cross-presented for the generation of HLA class I/peptide-complexes, allowing the induction of specific CTLs. However, some conflicting findings have been reported in the human, such as the lack of DCs maturation upon phagocytosis of apoptotic cells [17-19], so the fate and immunogenic potential of DCs that have internalized melanoma apoptotic cells or their debris remain an open issue. Several studies have used tumor cells virally transduced with TAAs or tumor cells apoptotized after infection with recombinant viruses encoding melanoma associated Ags [20-23] but few of these have evaluated the specific cross-presentation of native-melanoma Ags present in apoptotic tumor cells.

While we were writing this manuscript, Palucka et al [24] published the results of a phase I clinical trial of a vaccine composed of DCs loaded with killed allogeneic melanoma cells which induced objective clinical responses and elicited MART-1 specific CD8+T cells in stage IV patients. Thus, the use of a mixture of apoptotic/necrotic allogeneic melanoma cell lines as a complex source of melanoma Ags for DCs cross-presentation could be further explored to validate and complement these findings in the clinical setting.

The induction of apoptosis by different methods may produce a mixture of apoptotic/late apoptotic and/or necrotic tumor cells that could provide different signals necessary for DCs maturation as well as for CTL priming [4,25]. To investigate these points we produced and characterized human monocyte-derived DCs and co-incubated them with a mixture of four melanoma cell lines, rendered apoptotic/necrotic by γ radiation. Upon phagocytosis, we analyzed DCs maturation through the expression of surface markers and the decrease of the endocytic capacity, *in vitro *migration in response to chemokines and, most importantly, demonstrated their ability to cross-present native tumor Ags to specific CTLs *in vitro*.

## Methods

### Cell lines and clones

Four human melanoma cell lines were used in this study. Mel-XY1, Mel-XY2, Mel-XY3 and Mel-XX4 were cultured in melanoma medium (Dulbecco's Modified Eagle Medium: nutrient Mixture F12 (1:1) supplemented with 2 mM glutamine, 20 nM sodium selenite, 100 μM ascorbic acid, 0.3 mg/ml galactose, 0.15 mg/ml sodium pyruvate and 5 μg/ml insulin, 100 IU/ml penicillin, 10 μg/ml streptomycin) plus 10% fetal bovine serum (FBS) (Natocor, Córdoba, Argentina) in a GMP core facility at the Centro de Investigaciones Oncológicas-FUCA.

CTL clones (HLA A*0201 restricted) specific for MelanA/MART-1 (M27: AAGIGILTV) and gp100 (G154: KTWGQYWQV) Ags were expanded in RPMI medium in 14-day cycles by using 30 ng/ml anti-CD3 antibody (OKT-3, BD Biosciences) and serial 300 UI/ml IL-2 (Chiron BV, Amsterdam, Netherlands) every 3 days plus 10% heat-inactivated AB human serum and antibiotics [26]. Cell lines were periodically tested to be mycoplasma-free.

### Preparation of tumor apoptotic/necrotic cells

Apoptotic/necrotic tumor cells (Apo-Nec cells) were prepared as a batch of four cell lines from master cell banks after safety testing for mycoplasma, viruses and bacteria. The complete HLA haplotype of the four melanoma cell lines that compose Apo-Nec cells is: MEL-XY1: A*0201, A23; B18, B37; DR7, DR11, DR52, DR53; MEL XY2: A30, A33; B18, B65; DR1, DR1; MEL-XY3: A*0201, A23; B18, B18; DR11, DR13, DR52 and MEL-XX4: A24 (9), A33 (19); B18, B65 (14); DR1, DR11. All cell lines express Tyrosinase, MAGE 3, MelanA/MART-1 (except MEL-XX4 cell line), TRP-2, gp100, GD2, GD3 and NY-ESO-1 by RT-PCR and/or immunocytochemistry (unpublished results). After gamma irradiation at 70 Gy (Siemens Lineal Accelerator, Instituto Alexander Fleming, Buenos Aires, Argentina), the cells were frozen (50% DMEM, 40% human albumin and 10% DMSO) in liquid nitrogen until use. Cells were then thawed and plated in melanoma medium plus 10% FBS to complete the apoptotic process. After 72 hs the cells were detached from the flasks, washed, counted and resuspended in fresh serum-free AIM-V™ Medium (Therapeutic grade, GIBCO, Invitrogen Corporation, Grand Island, N.Y). Apoptosis and necrosis were assessed by Annexin-V and Propidium iodide (PI) binding (Annexin-V apoptosis detection kit, BD Biosciences, San José, CA) and Flow Cytometric (FACS) analysis (FACSCalibur, BD Biosciences, San José, CA). A soft agar clonogenic assay performed in sextuplicate (1.5 × 10^4 ^cells/well) was used to test that irradiated cells have lost their proliferation ability compared to non-irradiated control cells [27].

### Generation of DCs from monocytes

DCs were generated from buffy-coats or leukapheresis products obtained from healthy donors at the Hemotherapy Department of the Instituto Alexander Fleming. Peripheral blood mononuclear cells (PBMCs) were purified by Fycoll-Hypaque density centrifugation. PBMCs were resuspended in AIM-V medium and allowed to adhere to 0.22 μm filter-capped culture flasks (TPP, Germany). After 2 hs at 37°C, the non-adherent cells were removed, and adherent monocytes were subsequently cultured for 5 days in AIM-V supplemented with 800 U/ml rhuGM-CSF (kindly provided by Dr. Esteban Corley, PC-Gen, Buenos Aires, Argentina) and 50 ng/ml IL-4 (Peprotech, Mexico). Phenotypic changes were monitored by light microscopy and FACS. To induce control DCs maturation, 2 μg/ml LPS (Lipopolysaccharide from E. coli J5, Sigma, St. Louis, CA) was added and the cells were further cultured for 48 hs.

### DCs phenotype

Characterization of DCs phenotype was performed at the immature state (iDCs) and after Apo-Nec phagocytosis by staining 5 × 10^5 ^cells with fluorochrome-labeled antibodies (Abs) against CD14, CD11c, CD1a, HLA-DR/DP/DQ (HLA-II), CD80, CD86, CD83, CD40, HLA-ABC (HLA-I) and CCR7 (BD Biosciences, San Jose, CA), and FACS analysis was performed with CELLQuest software (Becton Dickinson, San Jose, CA). The corresponding isotype-matched controls used were FITC IgG1, FITC IgG2a and PE Rat IgG2a, (BD Biosciences, San Jose, CA.) Also, to analyze CD83 expression in double-positive PKH26/PKH67 DCs, we used an unlabeled anti-CD83 mAb (IgG1) and the corresponding isotype matched control, revealed with an anti-mouse IgG1-PerCP (BD Biosciences, San José, CA).

### FITC-dextran uptake

DCs endocytosis was evaluated by incubating 1 × 10^6 ^cells with 1 mg/ml FITC-dextran (FITC-Dx) (Sigma, St Louis, CA) for 30 min at 37°C. After washing with Phosphate Buffered Saline, cells were analyzed by FACS. Controls included tubes incubated with FITC-Dx at 4°C to inhibit the endocytic process and a basal uptake performed at 0 time point. Uptake was quantified by FACS analysis (10,000 cells per point).

### DCs phagocytosis of apoptotic/necrotic tumor cells

Apo-Nec cells (72 hs after irradiation plus culture) were co-cultured with iDCs (five days culture) at different ratios (1:1, 2:1 and 3:1) in fresh AIM-V medium for different time points. In some experiments DCs were dyed red with PKH26 and Apo-Nec cells were dyed green with PKH67-GL (Sigma, St. Louis, CA). After co-culture, FACS analysis was performed and DCs phagocytosis of Apo-Nec cells was defined by the percentage of double-positive cells. Appropriate controls were performed to set the cytometer for each color. A control for non-phagocytic binding of Apo-Nec cells to DCs was set by incubating the cells at 4°C for the same time points.

### *in vitro *DCs migration

DCs migration was assessed *in vitro *before and after co-culture with Apo-Nec cells, using a 48 wells chemotaxis chamber (AP 48 Neuroprobe Inc., Gaithersburg, MD). In the lower compartment, 10 ng/ml MIP-1α or MIP-3β (Peprotech, Rocky Hill, NJ, USA) were placed diluted in RPMI. Also, random migration was assessed placing RPMI in the lower chamber. DCs were seeded in the upper chamber (3 × 10^4 ^DCs/well) in RPMI. Between the upper and lower chamber a 5 μm pore polycarbonate membrane (Neuroprobe, Inc., Bethesda, MD) was placed. After 90 min at 37°C, the cells in the upper face of the membrane were scrapped out and the migrating cells adhered to the lower face of the membrane were stained with Giemsa. Membranes were air-dried, mounted onto a glass slide with Canadax and the cells were counted under the microscope. Five medium-power (400×) fields/well and 3 wells/condition were analyzed. Statistical analysis was performed using Student's t-Test.

### Electron microscopy

The phagocytic process was also studied by electron microscopy. Co-cultured samples were fixed at different time points with 2.5% glutaraldehyde in 0.1 M phosphate buffer pH 7.4, and then post-fixed in 1% OsO_4_, washed twice with distilled water and contrasted with 5% uranyl acetate for 2 hs. After washing and dehydratation, samples were embedded in Durkupan. Ultrathin slices (70–90 nm) were mounted in copper grids and contrasted with Reynold's lead citrate. Grids were analyzed under a transmition electron microscope Zeiss 109. Alternatively, to obtain whole-cell pictures, ultrathin slides (0.5 μm) were obtained in a ultramicrotome (Reichert-Jung), stained with 0.4% toluidine blue in 0.1 M carbonate buffer pH 7.4, mounted in Durkupan and analyzed under light microscopy (1000× magnification). Pictures were obtained with a Sony Cybershot Digital camera (5 megapixels) and images were further processed with Adobe Photoshop 6.0.

### *in vitro *cross priming assay – IFN-γ secretion

CD14+ monocytes were purified to 98% from a HLA A*0201 donor using anti-CD14 microbeads (Miltenyi Biotec, Germany) and were differentiated to iDCs by 5 days culture as described above. iDCs (6 × 10^5^) were loaded with Apo-Nec cells (2 × 10^5^) for 6, 12, 24 and 48 hs and incubated overnight with MelanA/MART-1 or gp100 specific CTL clones (2 × 10^5 ^cells) in 1 ml AIM-V medium. IFN-γ secretion to the supernatant was determined in triplicate by ELISA (OptEIA IFN-γ, Pharmingen BD Biosciences, San Diego, CA) according to the manufacturer's suggestions. A calibration curve was performed for each experiment and the sample concentration was calculated by log-log regression analysis using Cembal 2.2 software. Controls for this experiment included DCs loaded for 3 hs at 37°C with 20 μg/ml MART-1 or gp100 peptides plus 3 μg/ml β2-microglobulin, Ag-expressing live melanoma cell lines HLA A*0201 positive (positive controls) or DCs loaded with the non-specific peptides and Ag-expressing live melanoma cell lines HLA A*0201 negative (negative controls). G154 and M27 clones were also incubated with Apo-Nec cells and Apo-Nec Mel-XY2 (HLA*0201 negative, MelanA/MART-1 positive), and DCs alone.

### Measurement of intracytoplasmatic IL-10 and IL-12 cytokines

DCs were cocultured with Apo-Nec cells in a 3:1 ratio after labeling with PKH26 and PKH67 respectively, as described above. At 6, 12, 24 and 48 hs post coculture intracellular cytokines were accumulated by additional 8 hs-treatment with Brefeldin A (Golgi Plug, BD Biosciences, San José CA). After that, the cells were permeabilized with 0.05% saponin and stained with anti-IL10 (rat IgG2a)-APC and anti-IL-12 (p40–p70, mouse, IgG1)-PerCp (BD Biosciences, San José, CA). Isotype matched controls were also included. For FACS analysis the double-stained PKH26/PKH67 population was gated and the cytokines were evaluated in a four-color experiment. DC/Apo-Nec cells were compared to non-co-cultured DCs and Apo-Nec cells at each time point.

## Results

### Gamma-irradiation induced apoptosis of melanoma cell lines

**Figure 1:**
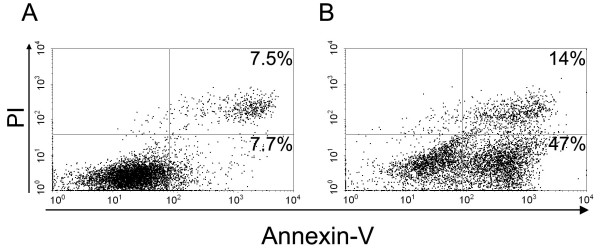
**Gamma irradiation of a mixture of melanoma cell lines induced apoptosis and necrosis (Apo-Nec cells)**. A mixture of four melanoma cell lines were cultured for 72 hs, either unirradiated (A) or after gamma irradiation at 70Gy (B) and stained with annexin V-FITC and PI. Early apoptotic cells were defined as annexin V-FITC^+^/PI^-^, while necrotic cells were double-positive. A representative experiment is shown.

We first tested exposure of the mixture of melanoma cell lines to different gamma irradiation doses (50, 70 and 100 Gy) to induce apoptosis. 50 Gy irradiation was enough to completely suppress the clonogenic capacity in the soft agar assay (0/15,000 colony/cells) for each melanoma cell line tested (not shown). When cells were irradiated at 70 or 100 Gy, no significant differences were observed in the degree of apoptosis/necrosis induced (data not shown). We have chosen to irradiate cells at 70 Gy and tested different incubation times after irradiation (24, 48, 72 and 96 hs) in order to complete the apoptotic process. In Figure 1 we observe that non-irradiated melanoma cells contained 6–9% early apoptotic cells characterized by Annexin-V^+^/PI^- ^staining (lower right quadrant). After irradiation at 70 Gy and 72 hs culture, 45–53% early apoptotic cells were obtained (Figure 1B, lower right quadrant). Necrotic cells stained with both Annexin-V and PI increased from 7.5% in non-irradiated cells to around 15% in irradiated cells, reflecting necrosis secondary to apoptosis (upper right quadrants). Thus, irradiated melanoma cells are defined as Apo-Nec cells in all the experiments that follow.

### Generation and characterization of DCs

To avoid deviation of the immune response to FBS-derived Ags, a serum-free culture protocol for *in vitro *generation of human DCs from PBMCs was developed. We obtained 5 × 10^6 ^DCs from 1 × 10^8 ^freshly plated PBMCs, after depletion of lymphocytes, which did not adhere to plastic culture flasks, in the presence of rhGM-CSF and IL-4. When cryopreserved PBMCs or leukapheresis products were plated under the same conditions, DCs yield was lower (3 × 10^6 ^DCs from 1 × 10^8 ^MN cells).

On day 5, aggregates of cells with the typical morphological features of DCs were harvested and phenotypically characterized. Control mDCs were obtained by adding 2 μg/ml LPS on day 5 and further incubating for 48 hs. Morphological changes during DCs maturation were observed by light microscopy and confirmed by FACS.

### iDCs efficiently phagocyte Gamma irradiation induced apoptotic/necrotic melanoma cells

**Figure 2:**
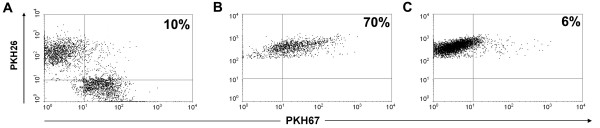
**DCs phagocytosis of Apo-Nec cells**. DCs were labeled red with PKH26 and Apo-Nec cells were labeled green with PKH67 and were analyzed by FACS without co-cultured (A). After coculture at 37°C for 48 hs at a 3:1 ratio (B) cells were analyzed by FACS. As a control cells was incubated for 48 hs at 4°C (C). The red population was gated and the double-positive phagocytic DCs were calculated as % in the upper right quadrants.

PKH26 red-labeled DCs were co-cultured with PKH67 green-labeled Apo-Nec cells (Figure 2A). After 48 hs phagocytosis was calculated as the percentage of double-positive cells, gated into the red-labeled population (DCs). At 37°C, 70% of DCs have phagocytosed Apo-Nec cells (Figure 2B) when they were co-cultured in a 3:1 DCs:Apo-Nec cell ratio. In these experiments non phagocytic Apo-Nec cells adherence to DCs was scarce since only 6% double-positive DCs were observed when labeled cells were co-cultured at 4°C for 48 hs to inhibit active DCs phagocytosis (Figure 2C). It is important to mention that although Apo-Nec cells were counted as entire cells, apoptotic bodies derived from the tumor cells were also present, representing two to three times the number of entire cells in the mixture (not shown).

**Figure 3:**
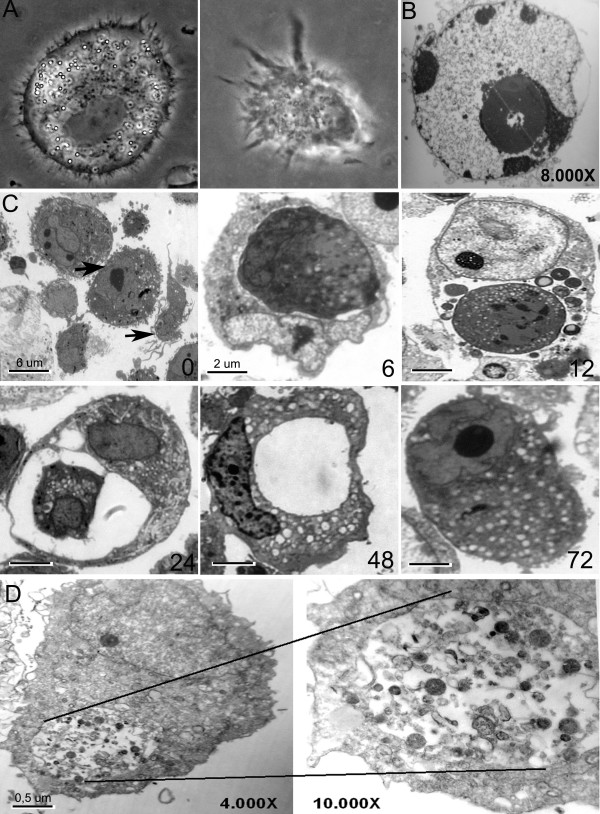
**Photomicrographs of DCs, Apo-Nec cells and the phagocytic process at different time points**. A – Characteristic morphologies of monocyte-derived iDC (left) and mDC (LPS-treated) (right) are shown under phase contrast microscopy (original magnification: 1000×). B – Electron microscopy of an apoptotic body (Apo-Nec sample) showing chromatin condensation; original magnification: 8000×. C – Representative pictures of the phagocytic process at 0, 6, 12, 24, 48 and 72 hs. In order to observe whole cells ultrathin slices (0.5 μm) stained by Toluidine blue were analyzed as described under methods. An Apo-Nec cell and a DC are indicated (upper and lower arrows, respectively). Original magnification: 1000×. D – Detail of a phagocytic DC observed under electron microscopy (4000×) showing digested material at higher magnification (10,000×).

Apo-Nec cells phagocytosis by iDC was further confirmed at different time points of co-culture in ultra-thin slices stained with toluidine blue and analyzed by electron microscopy. As shown in Figure 3, at 6 hs DCs had already engulfed Apo-Nec cells or apoptotic bodies and by 12–24 hs digested cellular material was seen inside large vacuoles. By 48–72 hs DCs returned to their normal size and empty residual vacuoles were frequently observed.

### Phagocytosis of apoptotic/necrotic melanoma cells induces DC maturation

**Figure 4:**
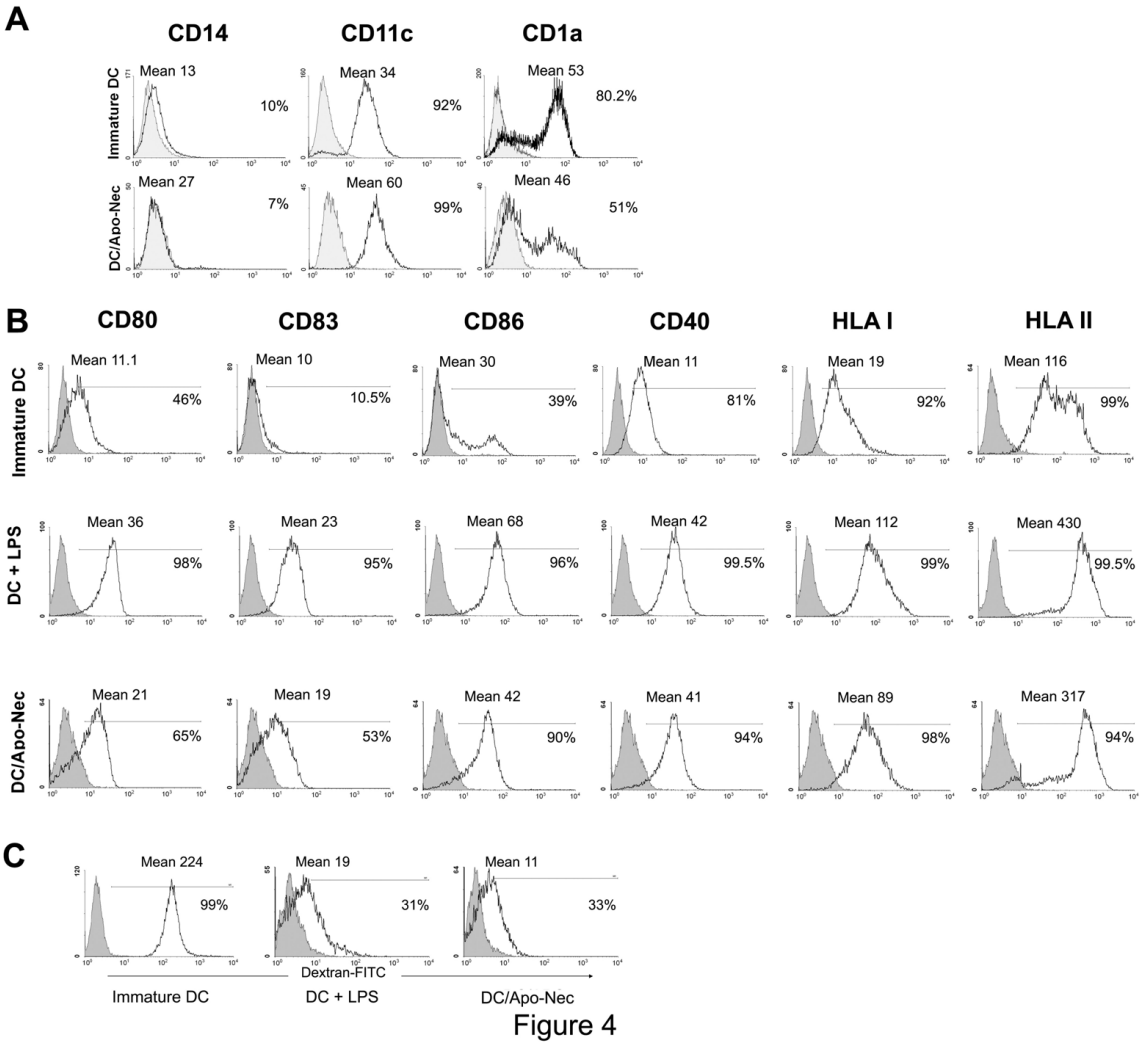
**Maturation of DCs after Apo-Nec cells phagocytosis**. A – DCs phenotype was studied before (iDCs) and after coculture with Apo-Nec cells (DC/Apo-Nec) analyzing CD14, CD11c and CD1a expression as described under Methods. In each case isotype-matched controls were performed. B – DCs marker expression of iDCs, LPS-treated DCs and DC/Apo-Nec. Cells were stained with FITC-labeled monoclonal antibodies as described under Methods and evaluated by FACS. Grey histograms represent the corresponding isotype matched control Abs. A representative experiment of six is shown. In each histogram the percentage of positive cells and the mean fluorescence intensity (Mean) are indicated. C – Endocytic activity of iDC, control LPS-treated DCs and DC/Apo-Nec were evaluated by the uptake of FITC-Dx as described under Methods. Percentage of endocytic DCs and Mean are indicated. A representative experiment of four is shown.

In order to stimulate naïve T cells, DCs must become mature increasing the expression of HLA Class I and Class II molecules and of co-stimulatory signals at the cell surface necessary to trigger T cell priming. iDCs and DCs co-cultured with Apo-Nec cells (DC/Apo-Nec) were phenotypically characterized by immunofluorescence. iDCs and DC/Apo-Nec were CD14^-^, CD11c+ and CD1a+ (Figure 4A). As observed in Figure 4B, phagocytosis of Apo-Nec cells induced DCs maturation similarly to LPS-induced DCs maturation, compared to iDCs. After 48 hs of Apo-Nec cells phagocytosis a marked increase in the expression of HLA class I and II, as well as of CD40, CD80 and CD86 co-stimulatory molecules on DCs was observed. Also, DCs maturation was evidenced by an increment in CD83 expression. In order to specifically analyze maturation of DCs that have phagocytosed Apo-Nec cells a three-color experiment was performed co-culturing red-labeled DCs (PKH26) with green-labeled Apo-Nec cells (PKH67) for 48 hs and then incubating the cells with PerCp labeled anti-CD83. When the double-positive PKH26/PKH67 population was gated, 55% of the cells were CD83+ (Mean: 40) compared to iDCs (13.8%, Mean: 22.8), indicating that DCs that have incorporated Apo-Nec cells are indeed up-regulating CD83 at the cell surface (not shown). Non-specific binding of antibodies to dying cells was ruled out in every experiment since anti-DCs markers binding to isolated Apo-Nec cells was less than 5% or absent (not shown).

DCs maturation was also evaluated by the decrease of DCs endocytosis after Apo-Nec cells phagocytosis (Figure 4C). FITC-Dx endocytosis was maximal in iDCS, 99 ± 5% uptake (Mean: 224), decreased to 31 ± 3% (Mean: 19) in LPS-maturated DCs and to 33 ± 5% (Mean: 11) in DC/Apo-Nec cells.

### DCs loaded with melanoma apoptotic/necrotic cells express CCR7 and migrate towards MIP-3 beta *in vitro*

**Figure 5:**
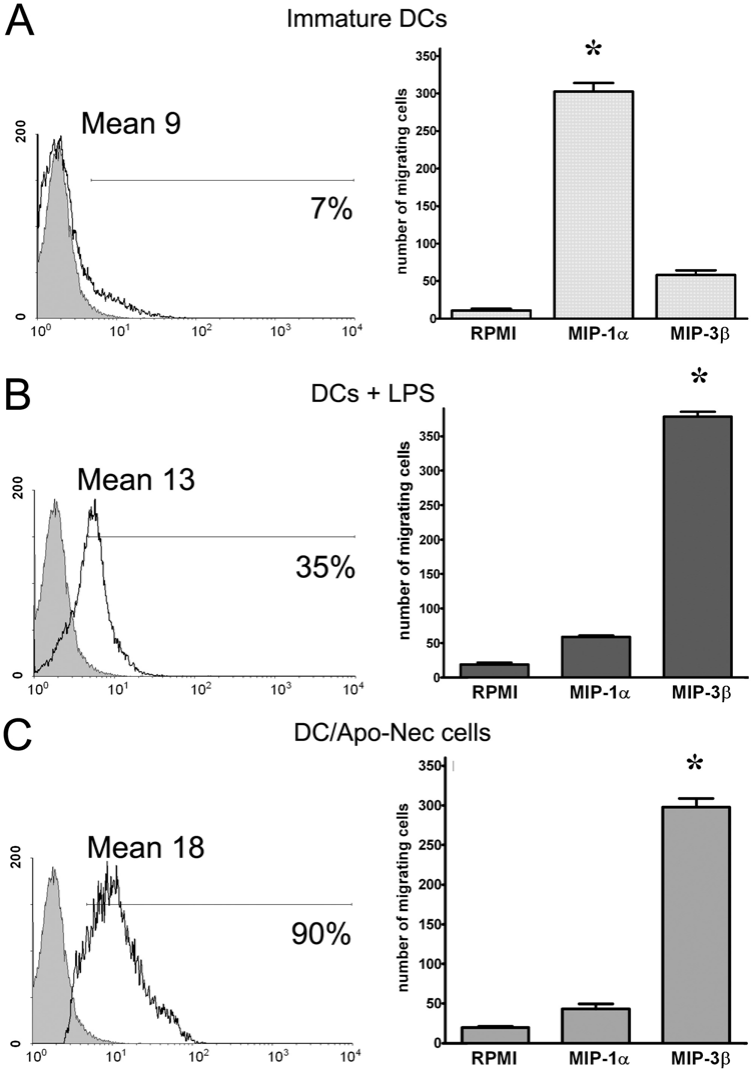
**CCR7 expression and *in vitro *DCs migration in response to MIP-1α and MIP-3β**. CCR7 expression (left panel) was analyzed by FACS in iDCs (A), LPS-treated DCs (B) and DC/Apo-Nec (C). Grey histograms represent the corresponding isotype matched control Ab. In each histogram the percentage of positive cells and the Mean are indicated. In vitro migration in response to MIP-1α and MIP-3β was evaluated (right panel) for iDC, LPS-treated DCs and DC/Apo-Nec cells as indicated. A control for random migration was done using RPMI medium. The number of migrating cells was calculated as the mean ± SD of cells counts in five medium-power (40×) fields/well performed in triplicate. A representative experiment of three is shown *p < 0.05 (Student's t-Test).

DCs migration to lymph nodes is crucial to trigger T lymphocyte priming. MIP-3β chemokine drives the homing of mDCs to the lymph node and interacts specifically with its receptor CCR7 expressed on the cell surface. iDCs expressed low levels of CCR7 (7%, Mean: 9) but increased its expression after Apo-Nec phagocytosis (90%; Mean: 18) or LPS maturation (35%, Mean: 13) (Figure 5). We observed that iDC migrated to MIP-1α but did not respond to MIP-3β (Figure 5A); on the contrary DC/Apo-Nec and DC + LPS migrated to MIP-3β but failed to respond to MIP-1α (Figure 5B and 5C). Thus, DC/Apo-Nec cells express MIP-3β receptor CCR7 and are able to migrate in response to MIP-3β, potentially allowing their homing to lymph nodes.

### DCs loaded with apoptotic/necrotic melanoma cells cross-present MelanA/MART-1 and gp100 Ags to specific T CD8+ cells

**Figure 6:**
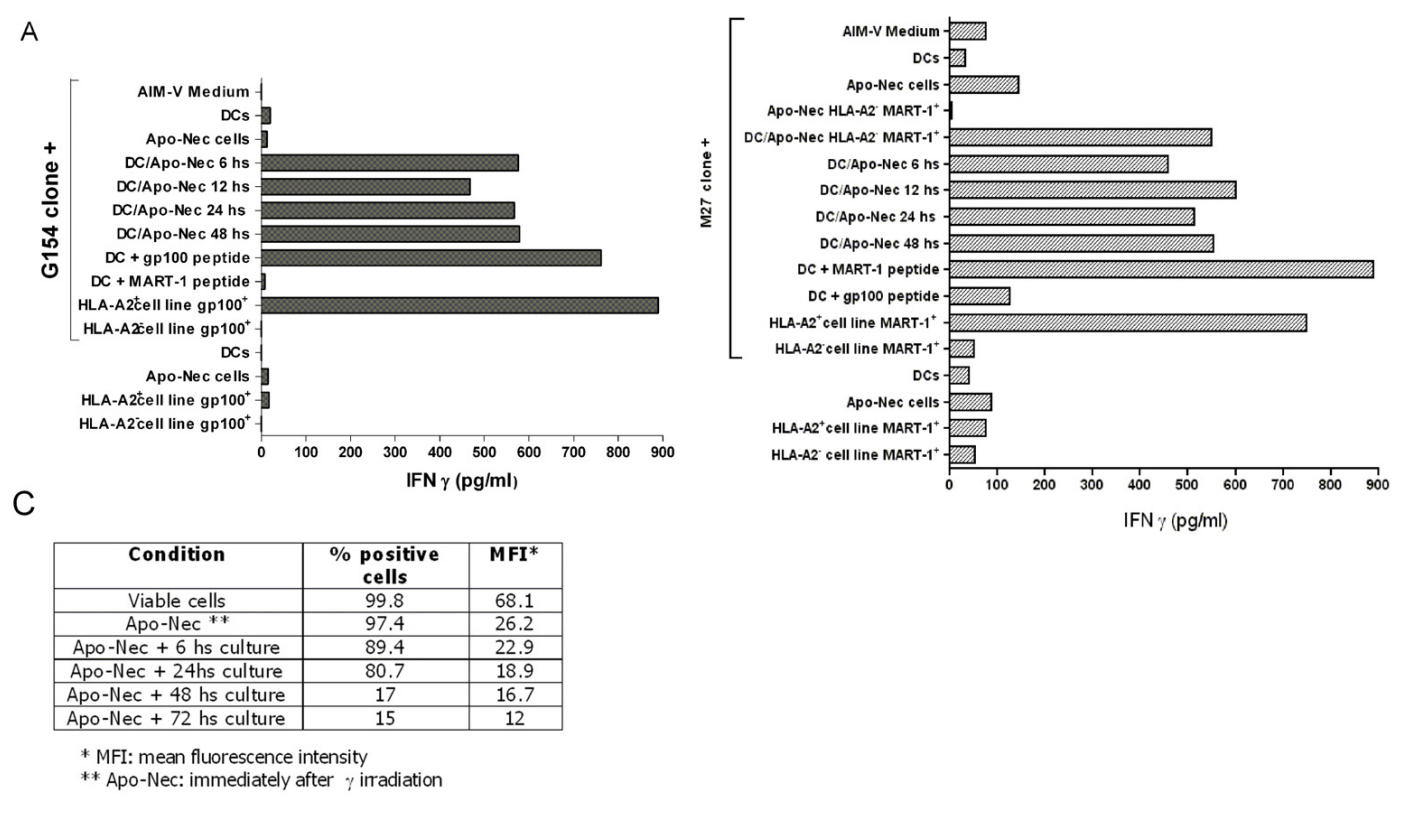
**DC/Apo-Nec cross-present MelanA/MART-1 and gp100 Ags to specific T CD8+ cells**. Overnight IFN-γ secretion from G154 clone specific for gp100 (A) and M27 clone for Melan A/MART-1 (B) Ags after different stimuli (indicated in the left side) was measured by ELISA as described under Methods. To allow cross-presentation for this experiment, DCs were obtained from an HLA-A*0201 donor and monocytes were purified by anti-CD14 magnetic separation as described under methods. For DC/Apo-Nec cells different time points after co-culture were studied. Results are the mean of triplicate determinations. (C) Expression of HLA-A*0201 in MART-1+ cell line (Mel-XY3) at different times after γ irradiation and culture was measured as described under Methods.

An important issue in this work was to assess if melanoma-associated Ags present in the Apo-Nec cells mixture (native Ags) could be cross-presented to specific CTLs after DCs phagocytosis. We analyzed IFN-γ secretion by specific CD8+T clones for MelanA/MART-1 (M27 clone) and gp100 (G154 clone) after overnight stimulation with DC/Apo-Nec cells. As observed in Figure 6A and 6B, DC/Apo-Nec co-cultured with the CTL clones efficiently induced IFN-γ secretion as soon as 6 hs and up to 48 hs after phagocytosis, evidencing cross-presentation for both Ags. For MelanA/MART-1 Ag we also observed IFN-γ secretion after incubation with DCs loaded with Apo-Nec HLA-A*0201 negative MelanA/MART-1^+ ^cells (Mel-XY2 cell line) clearly demonstrating cross-presentation for this Ag (Figure 6B). Controls for this experiment were performed, showing specific HLA-A*0201-restricted response using either DCs loaded with the corresponding peptides or CTL stimulation with live HLA-A*0201 positive gp100^+ ^MelanA/MART-1^+ ^melanoma cells (Mel-XY3) (Figure 6A and 6B) but lack of response using live MEL-XY2 cell line or with MelanA/MART-1 peptide for G154 clone or gp100 peptide for M27 clone. Mel-XY3 (HLA-A*0201 positive cell line) was rendered Apo-Nec by γ irradiation and also assayed for CTL priming, however, it only induced IFN-γ secretion by the CTL clones at short times after irradiation and culture (6–24 hs) (data not shown). After 48–72 hs of culture, when HLA-A*0201 positive Apo-Nec cells have completed the apoptotic-necrotic process, they failed to present both Ags to CTLs (Figure 6A and 6B, Apo-Nec bars). We observed that HLA-A*0201 haplotype expression was progressively lost in apoptotic/necrotic Mel-XY3 cells after gamma irradiation, only remaining 15–17% HLA-A*0201 positive cells (Mean: 16.7) 48–72 hs post irradiation (Figure 6C). Thus, 72 hs after irradiation Apo-Nec melanoma cells are no longer able to activate CTLs by themselves but may be used as a source of Ags for efficient cross-presentation after DCs phagocytosis and processing.

### Evaluation of Intracytoplasmatic IL-10 and IL-12

**Figure 7:**
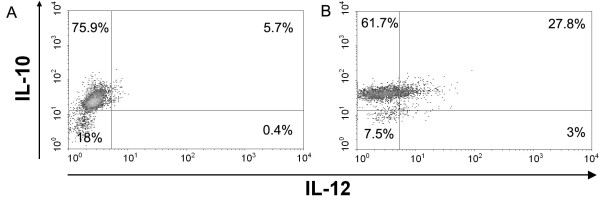
**Measurement of intracytoplasmatic IL-10 and IL-12 in DC/Apo-Nec cells**. In a four color experiment, intracytoplasmatic IL-10 and IL-12 cytokines were measured by FACS in iDCs (A) or DC/Apo-Nec (B) as described under Methods. Cells were labeled red (iDCs) and green (Apo-Nec cells) and after phagocytosis only the double-labeled population (DC/Apo-Nec cells) was analyzed with APC-anti IL-10 and PerCP IL-12 Abs after 8 hs treatment with Brefeldin A. 10,000 events per point were analyzed.

The balance between IL-10 and IL-12 in DC/Apo-Nec cells was quantitated by FACS at different time points after phagocytosis followed by 8 hs-treatment with Brefeldin A to accumulate intracytoplasmic cytokines. As shown in Figure 7, only 6.1% of iDCs produce IL-12 but after 32 hs of co-culture 30.8% of DC/Apo-Nec cells are induced to produce pro-inflammatory IL-12. Double-positive cells, producing IL-10 and IL-12, were 27.8% at 24 hs. After 56 hs of co-culture only 10% of the cells produced IL-12 (data not shown). For IL-10 instead, 81.6% of iDCs contained intracytoplasmatic cytokine and this did not significatively change after Apo-Nec cells phagocytosis. Apo-Nec cells were negative for both intracellular cytokines measured under the same experimental conditions.

## Discussion

There is now considerable experimental evidence that dying cells are capable of transferring Ags to the immune system for the induction of T cell immunity [19,28,29]. Albert et al first demonstrated in the murine model that apoptotic material could be processed and cross-presented by DCs to stimulate specific HLA-restricted CD8+ T cells [30]. In this study, the use of whole apoptotic/necrotic tumor cells to load DCs exploits both the advantage of maturation signals delivered by necrotic cells as proposed by Gallucci et al [31] and the optimal Ag processing and presentation in HLA class I and class II molecules by DCs [32] of a vast repertoire of known as well as yet unknown Ags from apoptotic cells for the induction of anti-tumor immune responses. Some controversy has arisen since several authors found that the uptake of pure or early apoptotic tumor cells did not induce proper DCs maturation [17,18]. However, these studies differ from the current one in several aspects: i) we used a mixture of four apoptotic/necrotic melanoma cell lines expressing several native melanoma-associated Ags instead of the single cell line approach or virally transduced tumor cells expressing exogenous Ags [21,22], ii) we used serum-free medium instead of FCS or autologous serum to generate monocyte-derived DCs [17], iii) the method used for tumor Ag preparation consists in gamma irradiation (70 Gy) and 72 hs culture in melanoma medium containing FBS as compared to higher energy radiation and culture in serum free-medium (producing mainly necrotic cells), UVB exposure or apoptosis induced by viral infection or by Fas pathway [17,33,34].

Previous reports [17,18] showed that early apoptotic melanoma cells or pure apobodies failed to induce mDCs, and either TNF-α, Poly I:C or cytokine cocktails were necessary to achieve DCs maturation and Ag presentation. In our case, phagocytosis of the mixture of melanoma cell lines used to load iDCs was enough to generate mDCs without addition of other stimuli. According to the results reported by Sauter et at [32] we took advantage of DCs maturation induced by necrotic tumor cells present in the mixture of Apo-Nec cell lines, as well as DCs ability of antigen processing and cross-presentation for CTL priming due to the apoptotic cells.

Using this particular melanoma cell lines mixture we wanted to address the question if the uptake of Apo-Nec cells could allow native TAAs to be processed to peptides by iDCs, mDCs and cross-present them to specific CTLs. As we have shown here, high levels of IFN-γ were secreted by M27 and G154 CTL clones after DC/Apo-Nec stimulation and, more importantly, M27 clone was also stimulated after incubation with DCs that have phagocytosed HLA-A*0201 negative MelanA/MART-1^+ ^gp100^+ ^Apo-Nec cells. This results clearly demonstrated that DCs have processed MelanA/MART-1 Ag taken up from the tumor cells and presented it to M27 clone in their own HLA-A*0201 context. As early as 6 hs after DCs loading with Apo-Nec, these cells could efficiently induce IFN-γ release, and we were able to measure CTL cross-presentation even 72 hs post DC/Apo-Nec co-culture. Several authors have identified gp100 as a regression Ag, since the induction of anti-gp100 immunity correlated with the regression of documented metastases in melanoma [25,35]. Besides, anti-MelanA/MART-1 CD8+T lymphocytes have also been detected in melanoma patients by tetramer staining and ELISPOT, correlating with clinical outcome and regressions [36]. Labarriere et al [18] reported that the use of purified melanoma apoptotic bodies to load DCs plus maturation with cytokines, efficiently cross-primed CTLs specific for NA-17A Ag but not for MelanA/MART-1 Ag. The authors could not detect MelanA/MART-1 epitopes in apobodies using a MelanA/MART-1 specific mAb. In our case, not only DCs matured after phagocytosis of Apo-Nec cells but the induction of IFN-γ secretion by a CTL clone specific for MelanA/MART-1 peptide was found.

Thus, our results suggest that a vaccine such as DC/Apo-Nec has the potential to initiate an immune response specific for MelanA/MART-1 and gp100 Ags and probably for other Ags expressed by these cells. Recently, Palucka et al [24]. have assayed in a phase I clinical trial a vaccine composed of DCs loaded with killed allogeneic melanoma cells demonstrating objective clinical responses and MART-1 specific CD8+T cell immunity. However, in this study the authors used a single HLA-A*0201 negative allogeneic melanoma cell line killed after a combination of TNF treatment, γ irradiation and culture in serum-free medium, plus the addition of CD40L to activate DCs. Our results further support the use of apoptotic/necrotic allogeneic tumor cells as a complex source of multiple melanoma native Ags to load DCs and show that a good maturation signal could be obtained with this particular mixture of melanoma cells, which also allows the cross-presentation of melanoma Ags to specific CTLs.

As we have demonstrated here, cross-presentation for MelanA/MART-1 and gp-100 Ags was achieved by DCs that have phagocytosed Apo-Nec cells but not by Apo-Nec cells themselves, since Apo-Nec cells (mixture) or HLA-A*0201 positive Apo-Nec cells (MEL-XY3 irradiated cells) were not able to induce INF-γ secretion separately. We have also observed that Apo-Nec cells progressively lost their HLA-A*0201 surface expression after irradiation and that their ability to present MelanA/MART-1 and gp100 peptides to CTLs decreased concomitantly (not shown). These results suggest that Apo-Nec cells are not presenting the peptides to CTLs by themselves but, after their phagocytosis, processed peptides are being cross-presented to CD8+ T cells by DCs. A recent report by Blanchére et al. [37] suggested in a murine model that apoptotic cells may be critical in processing Ags for cross-presentation, in essence by pre-selection of immunologically important antigenic determinants. In this view, our results in the human setting further support this hypothesis, since tumor dying cells (mainly apoptotic cells) can be used as a source of processed tumor determinants for DCs loading and cross-presentation to CTLs. Furthermore, presentation by DCs of Ags generated in apoptotic melanoma cells has the potential benefit that presentation via HLA class II may generate helper epitopes that support the development of specific CD4+ lymphocytes that might be important for antitumoral immunity. We cannot address if Ag peptides are being processed into Apo-Nec cells and then taken up by DCs and presented in the HLA class I context or if DCs have processed them after Apo-Nec phagocytosis. Besides, tumor-derived exosomes loaded onto DCs have been shown to trigger MART-1 melanoma Ag cross-presentation to specific CTLs [38] Although we used washed Apo-Nec cells resuspended in fresh AIM-V medium in all experiments and a differential ultracentrifugation of culture supernatants is required to obtain tumor-derived exosomes, we cannot exclude the contribution of exosomes that might be released by Apo-Nec cells during the 48 hs co-culture with DCs. Nevertheless, our main objective has been to assess if this particular mixture of Apo-Nec cells (either entire or their fragments/debris after gamma irradiation) was able to be phagocytosed by iDCs, induce iDCs maturation, migration and cross-presentation of native tumor peptides to specific CD8+ T cells.

We have also evaluated DC/Apo-Nec cells migration to MIP-3β as a measure of their potential homing to lymph nodes. Upon phagocytosis, DCs must reach the lymph nodes in response to chemokine concentration gradients such as MIP-3β in order to prime naïve T cells. It was important to asses if DC/Apo-Nec could respond *in vitro *to MIP-3β. We found that like fully mature LPS-treated DCs, DC/Apo-Nec cells up-regulated MIP-3β receptor (CCR7) and efficiently migrated *in vitro *to MIP-3β but not to MIP-1α. Our results are coincident with those reported by Hirao et al, who found specific DCs migration to MIP-3β *in vitro *and *in vivo *and CCR7 induction after phagocytosis of UV-treated fibrosarcoma cells [39].

The production of the pro-inflammatory cytokine IL-12 requires two signals: IFN-γ and a maturation signal provided by CD40 ligation (CD40L) or LPS. Recently, Xu et al [39,40] have proposed that Toll – like receptor 8 provides a priming signal for high production of IL-12. Production of IL-12 and IL-10 influences DCs maturation and the induction of a potent immune presentation to T cells [40,41]. Accordingly, we found that upon phagocytosis of Apo-Nec intracellular pro-inflammatory IL-12 transiently increased while IL-10 did not change in DC/Apo-Nec cells.

We believe that our results complement the existing reports about the use of apoptotic and necrotic cells to load iDCs, and demonstrated that they induce DCs maturation, up-regulate CCR7 allowing their migration to MIP-3β, and efficiently process native melanoma Ags and cross-present them to specific CTLs. This particular mixture of apoptotic/necrotic cell lines phagocytosed by iDCs should be tested as a vaccine for melanoma patients since it could provide mature melanoma Ag-loaded DCs for efficient cross-priming to elicit anti-tumor immunity.

## Conclusion

We used a mixture of four melanoma apoptotic/necrotic cell lines as a source of native Ags to load human monocyte-derived iDCs. After phagocytosis, we found that Apo-Nec cells induced DCs maturation without addition of further stimuli, increased *in vitro *migration in response to MIP-3β chemokine and intracellular IL-12 production. Most importantly, we demonstrated the ability of DC/Apo-Nec cells to cross-present native tumor Ags to Ag-specific CTLs *in vitro*. We suggest that our results with DC/Apo-Nec should be explored as a vaccination strategy in melanoma patients.

## Competing interests

The author(s) declare that they have no competing interests.

## Authors' contributions

EMvE carried out the DC-loading and migration experiments, developed the cross-presentation studies and interpretation of data.

MMB participated in the design, analysis of the experiments and drafted the manuscript.

DF assisted with CTL clones culture.

MB helped with figures preparation.

EL, RW participated in the discussion of the results.

YQL established and characterized the CTL clones.

CY provided the CTL clones for assessment of DC/Apo-Nec cross-presentation.

JM conceived the study and participated in its design and coordination and helped to draft the manuscript.

All authors read and approved the final manuscript.
